# Home-Based Rehabilitation Following Subtalar Dislocation: A Case Report

**DOI:** 10.7759/cureus.41348

**Published:** 2023-07-04

**Authors:** Eugénio Moita Gonçalves, Belmiro Alves, Francisco Antunes, José Vítor Gonçalves, Catarina Aleixo, Daniel Vilaverde, Filipe Maçães, Rodrigo Correia, Inês Andrade, Andreia Ferreira

**Affiliations:** 1 Physical Medicine and Rehabilitation, Vila Nova de Gaia/Espinho Hospital Center, Porto, PRT; 2 Research, Faculty of Medicine, University of Porto, Porto, PRT; 3 Research, Egas Moniz Health Alliance Clinical Academic Center, Aveiro, PRT; 4 Orthopedics and Traumatology, Vila Nova de Gaia/Espinho Hospital Center, Porto, PRT; 5 Medical Imaging, Vila Nova de Gaia/Espinho Hospital Center, Porto, PRT; 6 Physical Medicine and Rehabilitation, Vila Nova de Gaia/Espinho Hospital Center, North Rehabilitation Center, Gaia, PRT; 7 Physical Medicine and Rehabilitation, Alcoitão Rehabilitation Medicine Center, Lisbon, PRT

**Keywords:** case report, self-rehabilitation, subtalar dislocation, subtalar joint, home-based rehabilitation

## Abstract

The demand for physical medicine and rehabilitation services has risen significantly. Immediate rehabilitation is not always readily available which may compromise patients' functional recovery. Here, we describe a rare subtalar dislocation case and how an unsupervised home-based rehabilitation program allowed functional recovery.

A 49-year-old male presented to the emergency department with an injury to the right ankle which resulted from a 3-meter height fall with his foot in plantar flexion and inversion. Clinical and imaging findings confirmed a diagnosis of a rare case of subtalar dislocation. The post-injury AOFAS Ankle-Hindfoot Scale score was 24/100 points. After six weeks of immobilization, a patient-tailored home-based rehabilitation program was prescribed. Adherence to our home-based rehabilitation program was critical to allow a range-of-motion improvement and functional recovery.

Delaying rehabilitation may lead to long-term functional impairments. Thus, acknowledging the post-acute period as critical to initiate rehabilitation is mandatory. When outpatient rehabilitation settings are not readily available due to high demand, comprehensive patient education, and home-based rehabilitation programs may constitute effective alternative interventions. We demonstrate the significant improvement obtained with an early patient-tailored home-based rehabilitation program in range-of-motion and functional outcomes in a case of medial subtalar dislocation.

## Introduction

The demand for physical medicine and rehabilitation services has been on a significant rise across a multitude of countries with varying levels of income, due in part to the rise in total disability-adjusted life years related to populations’ life expectancy increase [1]. National health systems, including Portugal’s, are still severely under-resourced regarding their rehabilitation network since it is often not possible to assure timely rehabilitation integration following specific patient diagnoses, especially acute traumatic injuries [1]. The following subtalar dislocation case report reflects this imminent reality.

Subtalar dislocations are extremely rare injuries that comprehend 1% of all traumatic injuries to the foot and are described as simultaneous dislocation of the talocalcaneal and talonavicular joints without any tibiotalar or talar neck-associated fractures [2]. The medial variant represents the most common form (85%) and usually results from high-kinetic energy trauma involving an inversion force directed to the subtalar joint. In the event of these dislocations, a promptly closed reduction under sedation should be performed to avoid further soft tissues and neurovascular damage, which will then be followed by a period of four to eight weeks of cast immobilization to assure soft tissue healing, as discussed by Lasanianos and colleagues. As supported by the literature, after cast removal, early rehabilitation is of paramount importance in order to achieve the best functional results [2]. However, following orthopedics referral, early supervised outpatient rehabilitation is not readily available or even feasible. Thus, home-based exercise programs may constitute a cost-effective early intervention that allows patients to maximize specific rehabilitation goals ahead of integration into a delayed rehabilitation program.

Here, we describe how an unsupervised home-based rehabilitation program was implemented for a case of medial subtalar dislocation without fracture which improved functional outcomes ahead of a supervised program in a high-demand outpatient rehabilitation setting. The reported results support our hypothesis that implementing home-based rehabilitation programs could assist in alleviating the strain on outpatient rehabilitation services.

## Case presentation

A 49-year-old physically active deaf male presented to the emergency department, accompanied by his mother, with an injury to the right ankle. The injury resulted from a 3-meter height fall from a tree with his right foot in a maintained position of plantar flexion and inversion causing pain, significant deformity, and disability. The patient had an unremarkable medical and surgical history, particularly in regard to ankle sprains or ligament laxity until this event.

**Figure 1:**
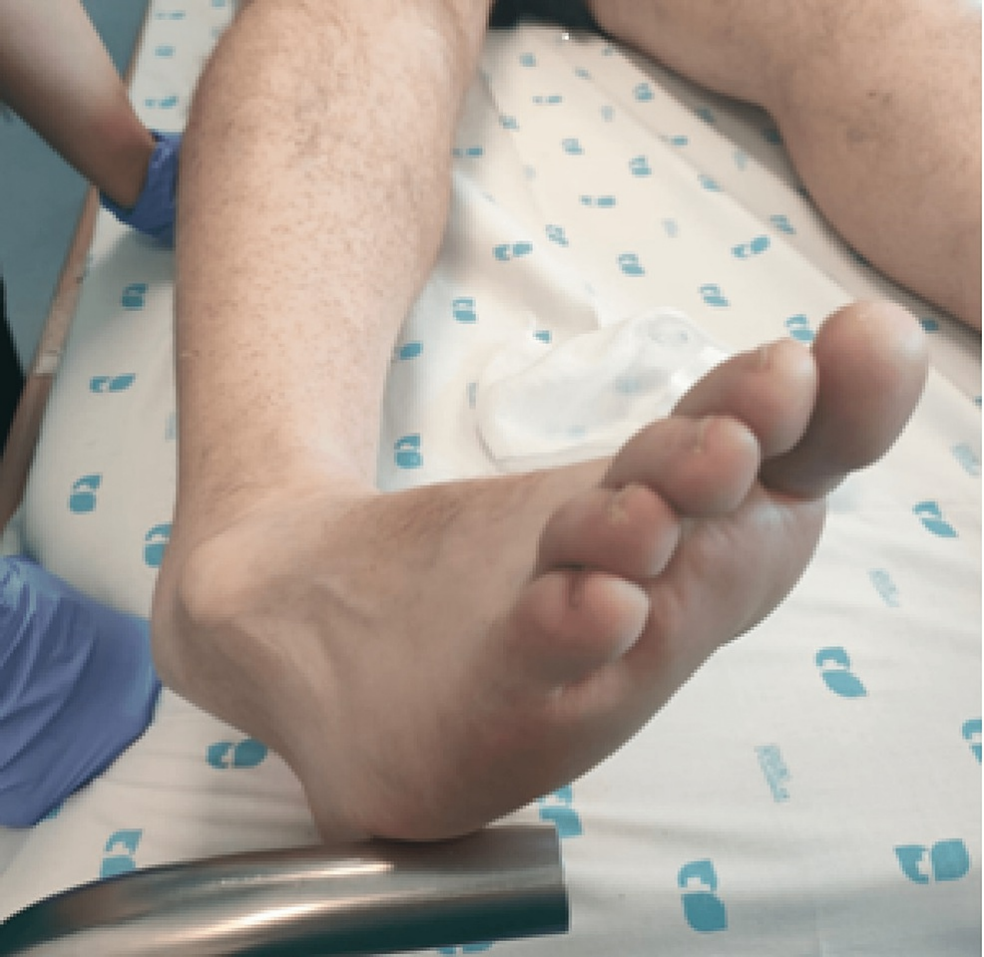
Photograph showing hindfoot medial displacement

**Figure 2:**
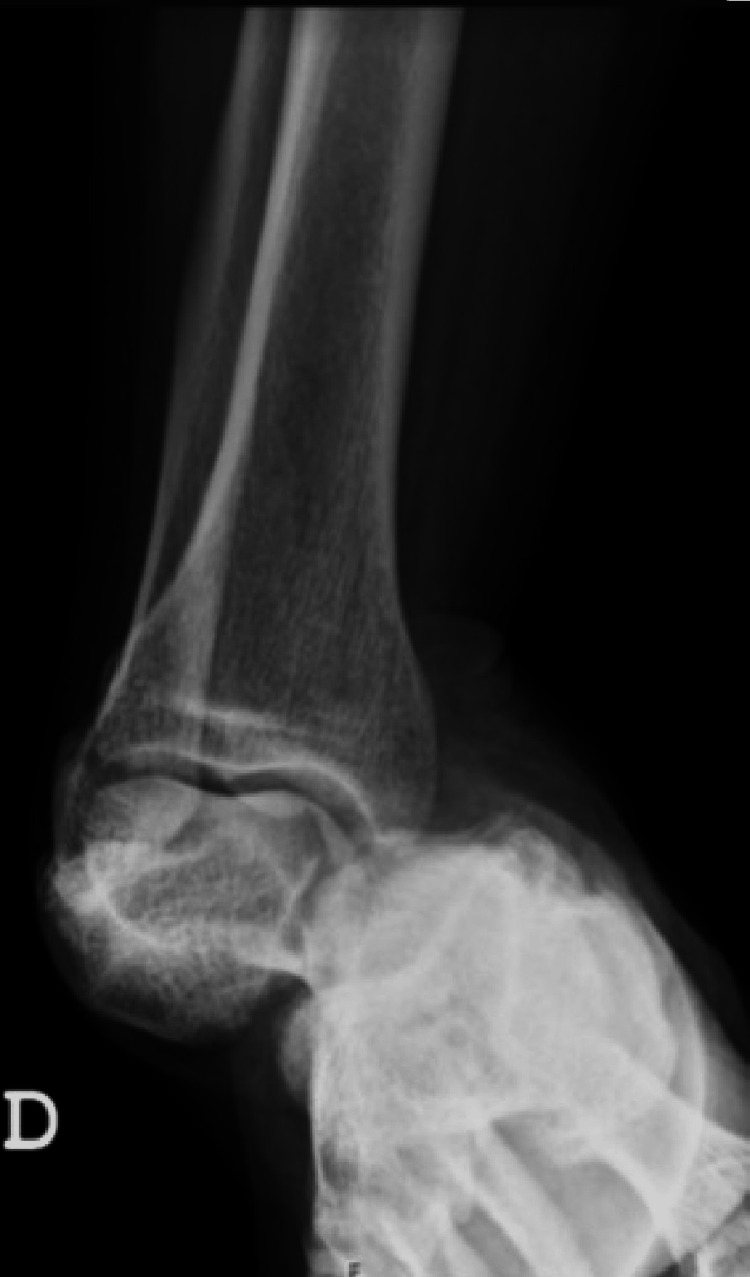
Anteroposterior radiographic view showing dislocation of the talonavicular and talocalcaneal joints without fracture.

On arrival, the emergency orthopedic team noted a severely deformed and medially displaced foot with the prominent head of the talus projected dorsolaterally, just below the lateral malleolus (Figure 1). Although the skin over this anatomical landmark was stretched, there was no evident swelling, wounds, or ecchymosis. No neurological deficits were documented. The affected extremity was warm, except for the toes (capillary refill time > 3 seconds), and the dorsalis pedis pulse and the posterior tibial pulse were not easily palpable. An early radiograph confirmed a talocalcaneal and talonavicular joint dislocation, without evident fracture (Figure 2). Calcaneus and navicular bone were dislocated medially in relation to the ankle mortise in the anteroposterior view.

After an unsuccessful early attempt to correct the deformity position, the patient was referred to the emergency room for close reduction with sedoanalgesia and sciatic nerve block, which was achieved at the first attempt, with optimal stability. Axial manual traction on the foot and heel in the line of the deformity was combined with countertraction with the knee in 90º flexion to relax the triceps surae complex pull over the calcaneus. An eversion force was applied on the foot with the ankle in plantar flexion followed by dorsiflexion. Dorsalis pedis and posterior tibialis pulses returned to be easily palpable. After applying a below-knee cast for tissue healing, a new radiographic image confirmed successful reduction with accurate joint alignment. Additionally, a computed tomography (CT) scan was conducted, revealing the presence of heterotopic calcifications located on the anterior talofibular ligament.

**Figure 3:**
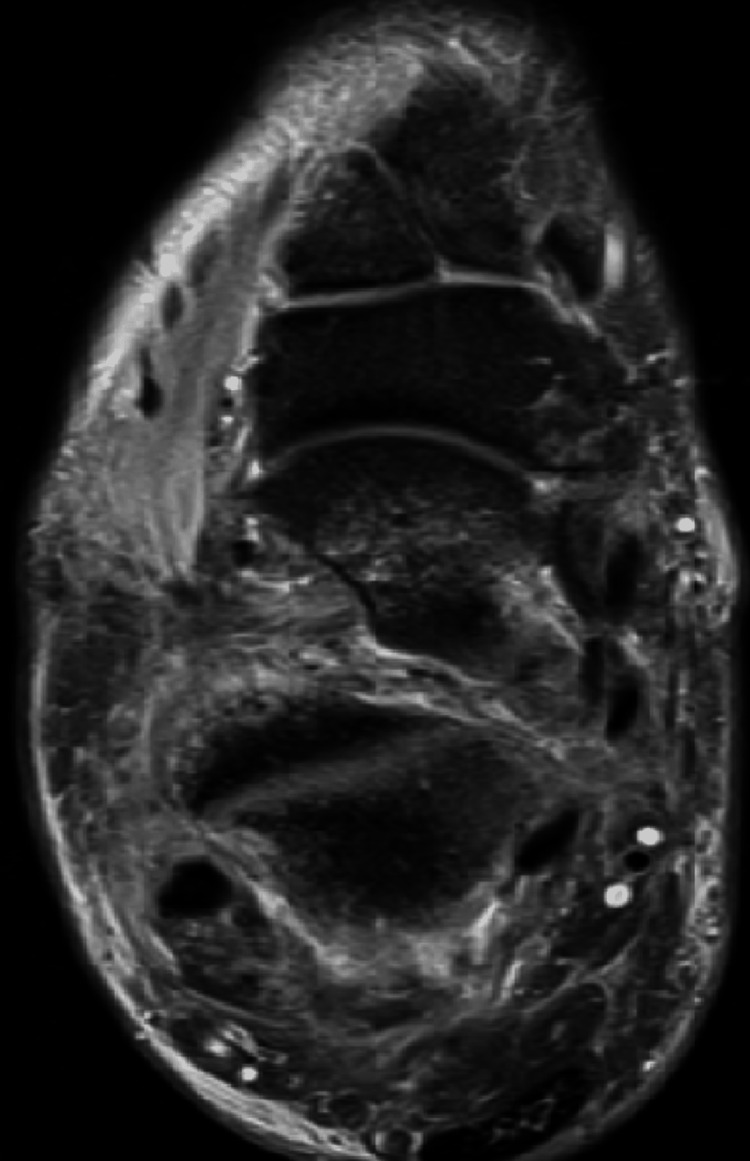
Diffuse soft tissue edema in the tarsal sinus, midfoot and rearfoot regions.

**Figure 4:**
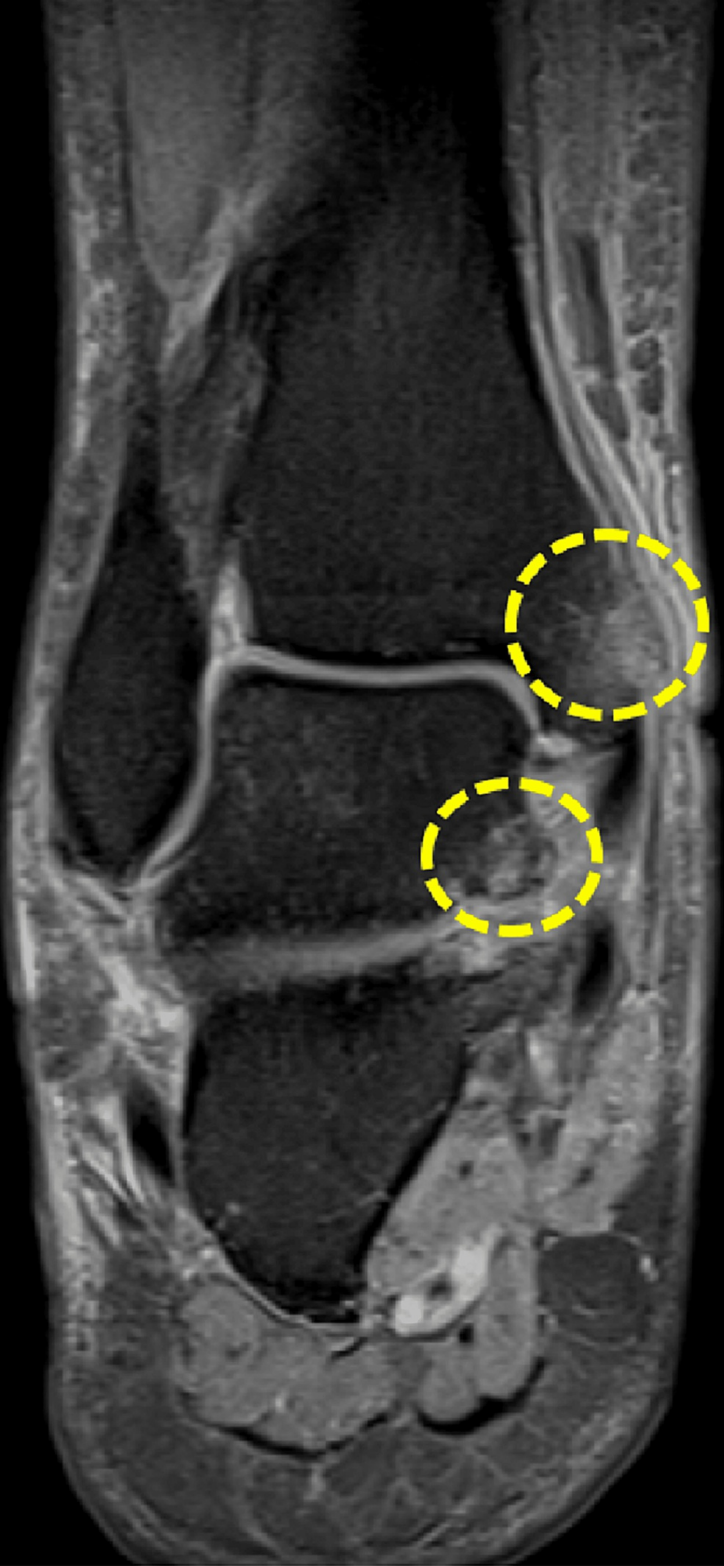
Contusional edema of the medial malleolus and the medial process of the talus.

**Figure 5:**
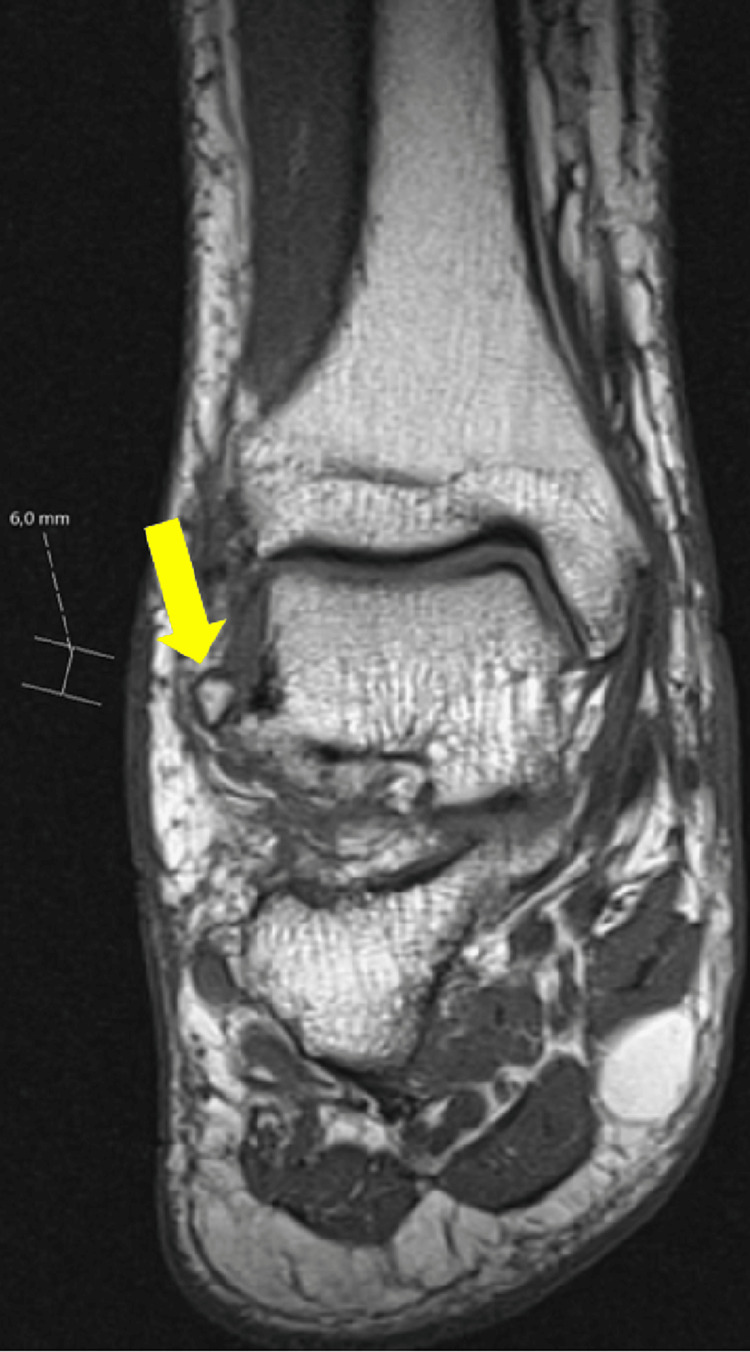
A 6-mm osseous fragment of avulsive nature coexists anteriorly to the distal epiphysis of the fibula.

**Figure 6:**
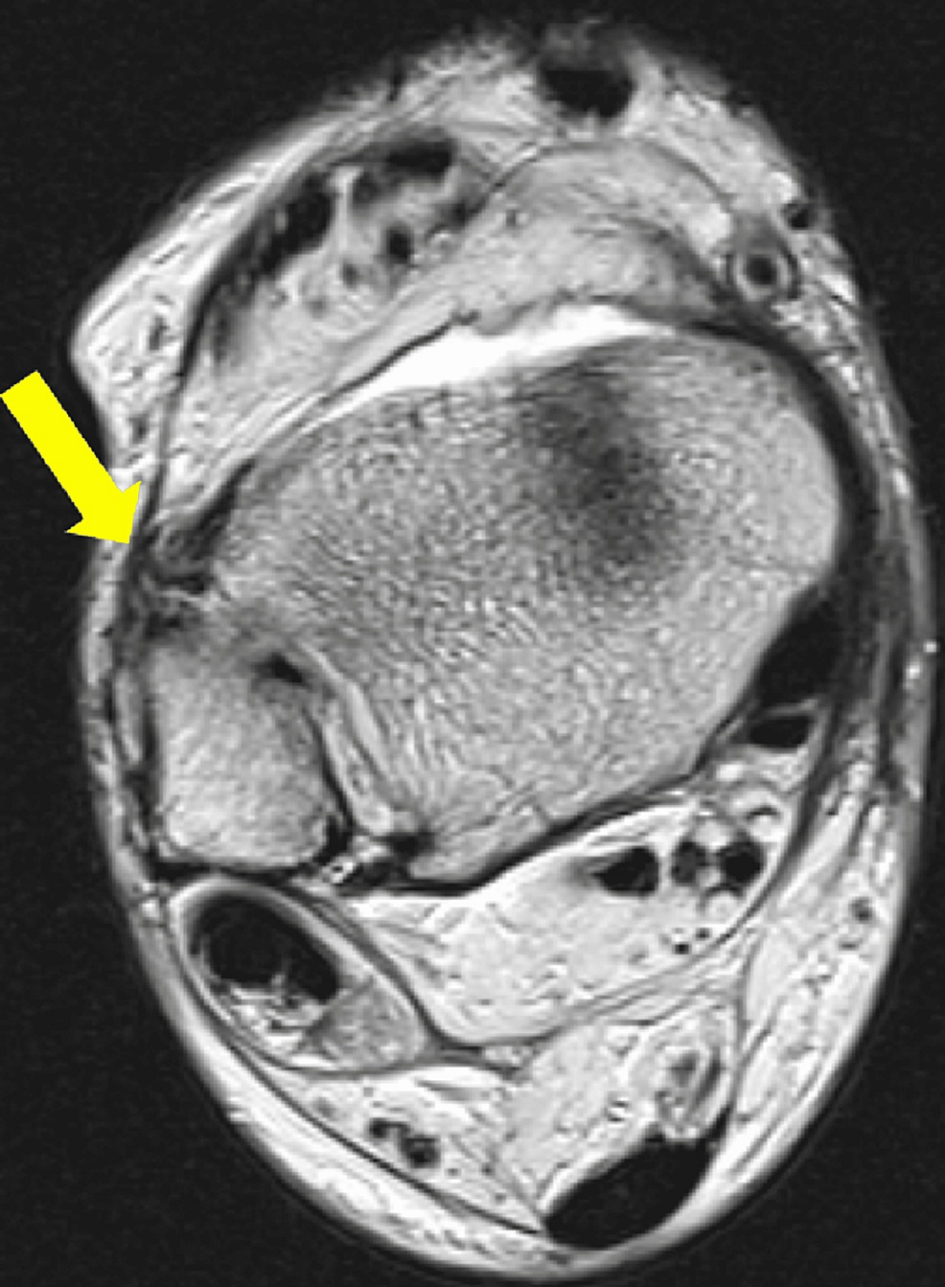
Thickening of the anterior syndesmosis is identified, suggesting a focal discontinuity (arrow), likely corresponding to a complete thickness rupture (grade 3).

**Figure 7:**
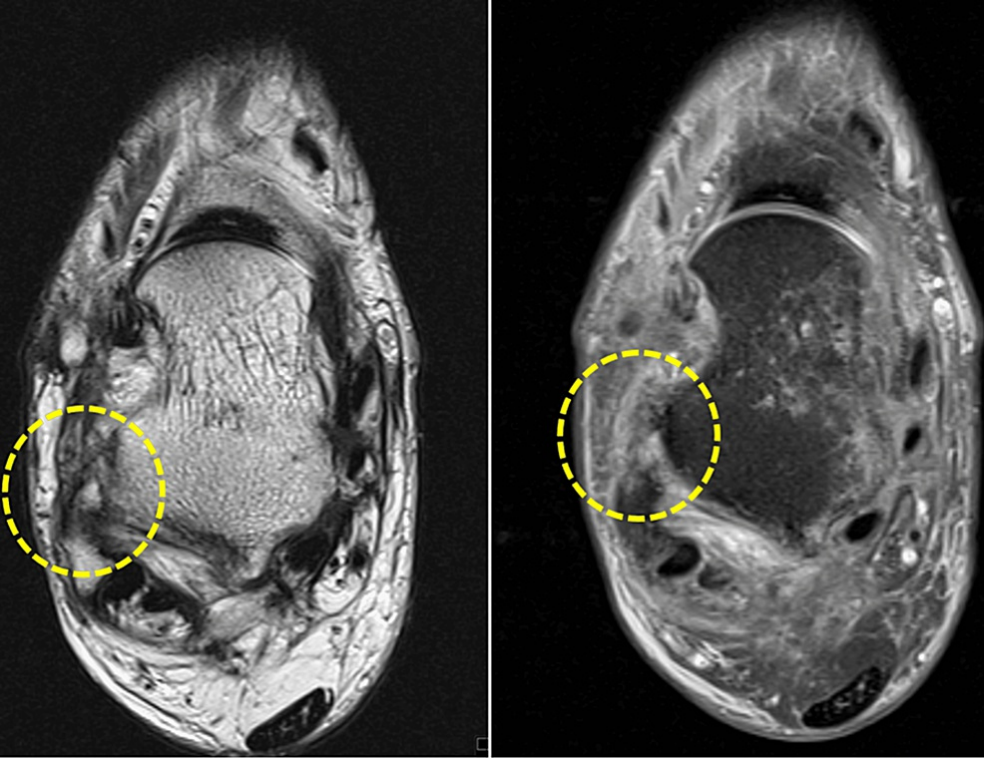
Thickening and architectural distortion of the anterior talofibular ligament are observed, indicating a grade 2 rupture.

**Figure 8:**
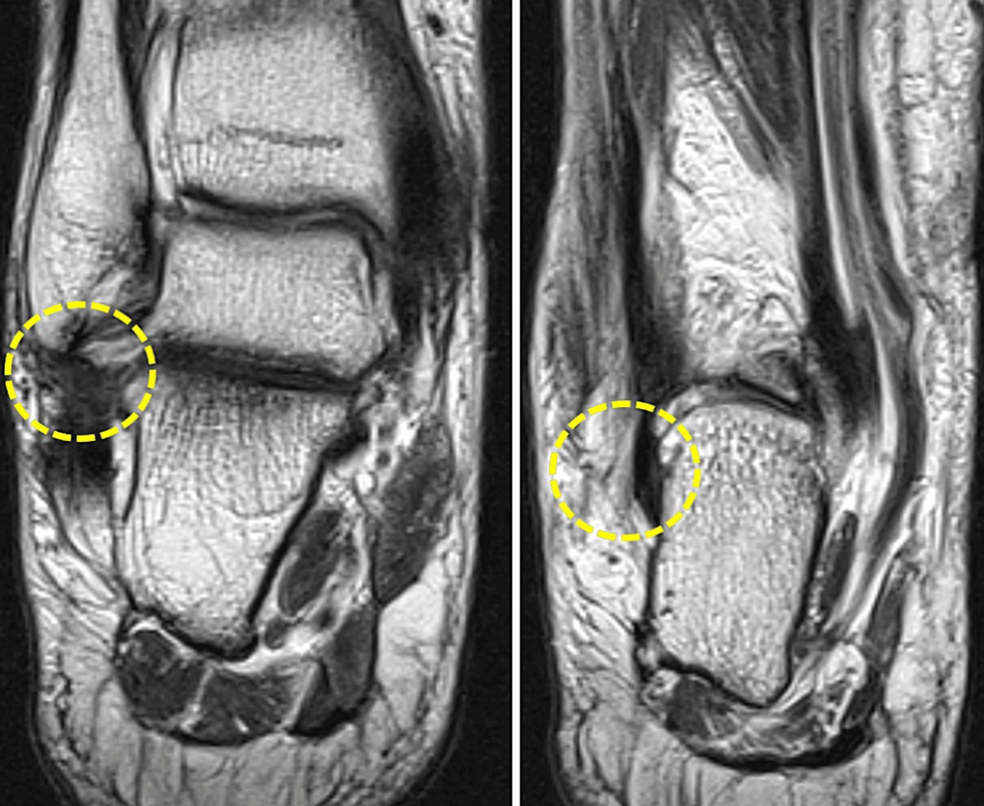
Thickening and edema of the calcaneofibular ligament are observed, primarily in its proximal third (grade 2 rupture), and to a lesser extent, at its distal insertion on the peroneal tubercle of the calcaneus.

Subsequently, the patient was released from the hospital with instructions to uphold a non-weight bearing status until the scheduled follow-up appointment with outpatient Orthopedics, which was set for the sixth week post-injury. During this appointment, a reassessment would take place. Thus, following a period of six weeks of immobilization, the cast was removed. At the time, on physical examination, the patient had moderate hindfoot pain with pressure, mild swelling on the ankle, and significant subtalar joint range of motion (ROM) limitation. The American Orthopedic Foot and Ankle Society (AOFAS) Ankle-Hindfoot Scale score, comprising assessments of pain, function, and alignment, yielded a total of 24 out of 100 points. Radiographic findings did not demonstrate any indications of arthritis or avascular necrosis affecting the talus. In order to obtain a more comprehensive understanding of the injury, an MRI scan was performed, revealing the presence of various ligamentous tears, encompassing the tibiofibular syndesmotic ligaments, the anterior talofibular ligament, as well as the talocalcaneal ligaments. Furthermore, the MRI also detected partial ruptures in the tendons of the tibialis posterior and peroneus brevis (Figures 3-8).

**Figure 9:**
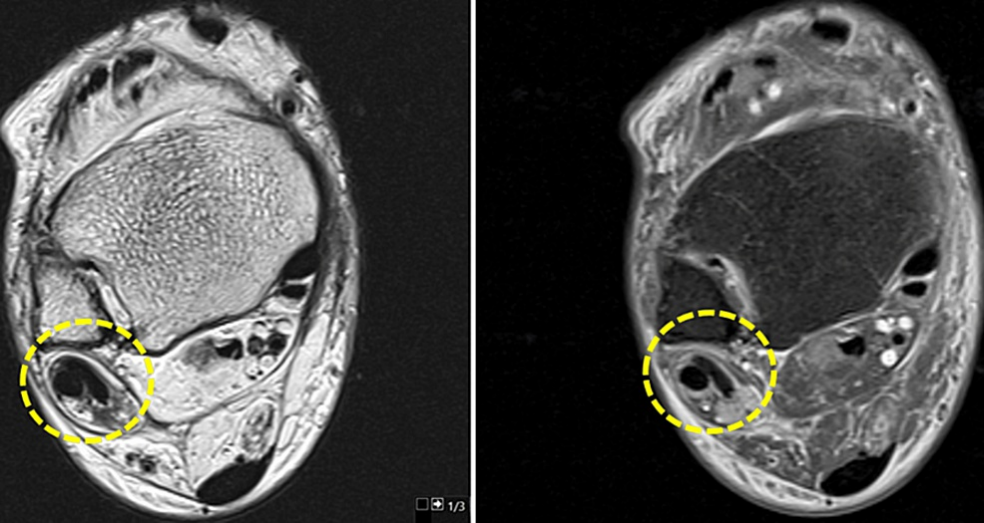
Cashnew nut deformity of the peroneus brevis tendon is identified, indicating a partial “C-shaped” longitudinal rupture caused by compression between the peroneus longus tendon and the lateral malleolus in dorsiflexion.

**Figure 10:**
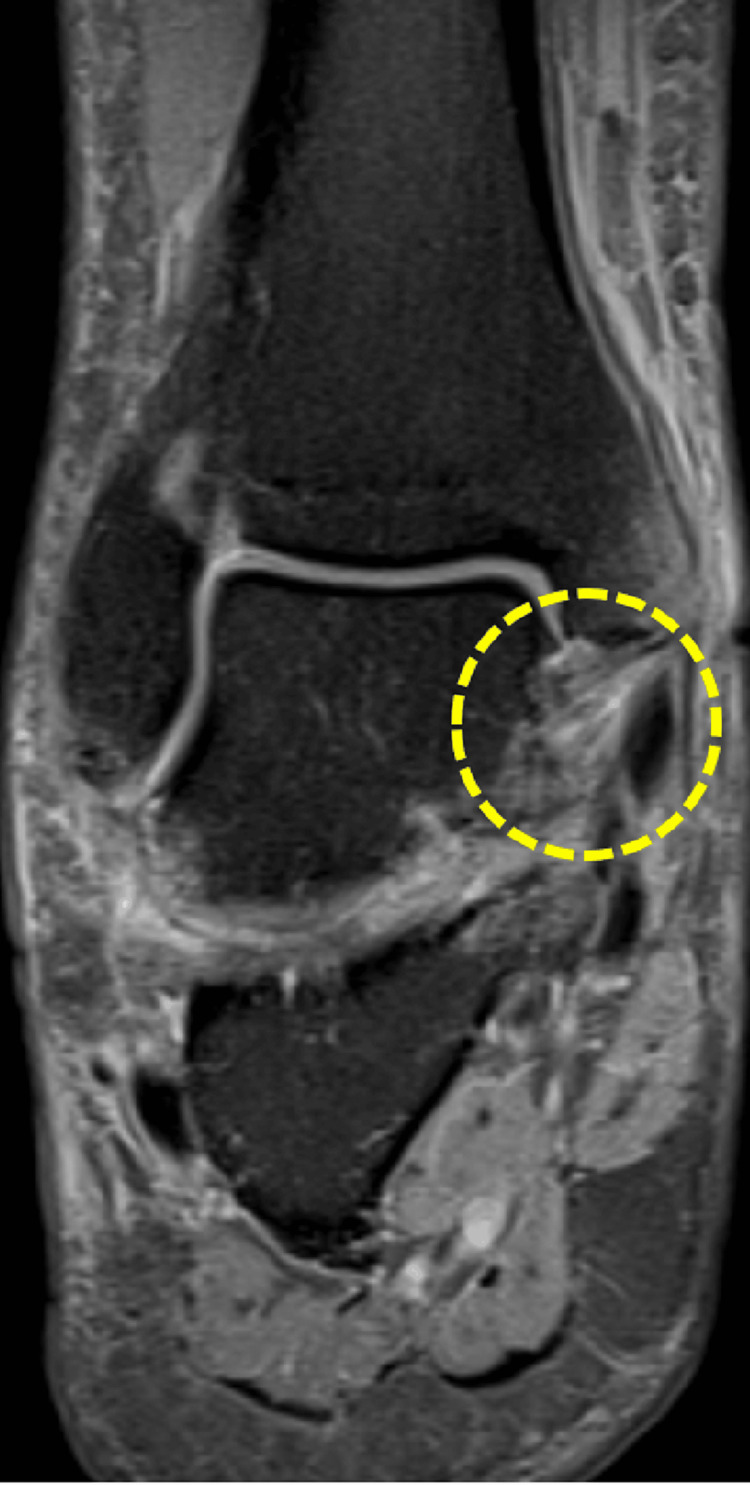
Edema of the deltoid complex is observed, secondary to the injury, without significant architectural distortion of its fibers (grade 1 injury).

Subsequently, the patient received detailed instructions to commence partial weight bearing and was prescribed a patient-tailored home-based exercise program provided in the form of a booklet. The prescribed program encompassed various components, including ankle joint flexibility exercises involving ROM movements for foot dorsiflexion, plantar flexion, inversion, and eversion. Additionally, the program included strengthening exercises such as eccentric Achilles strengthening, heel raises, concentric ankle dorsiflexion, eversion, and inversion exercises utilizing an elastic band and towel scrunches. Furthermore, proprioception exercises comprising single leg taps and balance training using a pillow on the ground were also incorporated. The prescribed exercise regimen elucidated the recommended number of repetitions, sets, and frequency per day for each exercise. Instructions on employing static cryotherapy were provided as a means to manage pain or any indications of inflammation. Prior to initiating the exercises at home, the patient also received comprehensive guidance on the correct execution of each exercise and was encouraged to adhere to the program consistently.

During the eighth week following the injury, the patient underwent a comprehensive clinical and functional evaluation at a Physical Medicine and Rehabilitation appointment. At this stage, the patient had progressed to full weight-bearing mobilization with the assistance of an ankle brace for compression and control of dorsiflexion and plantar flexion. Ankle ROM measurements using a goniometer revealed 10º of dorsiflexion, 20º of plantar flexion, 15º of inversion, and 5º of eversion. The AOFAS Ankle-Hindfoot Scale indicated a score of 72. The patient expressed adherence to the home-based exercise program, describing the proper execution of exercises and reporting significant improvements in activities of daily living (ADL) participation with minimal pain.

Subsequently, a personalized physical and functional rehabilitation program was formulated by the physiatrist. The program aimed to optimize the subtalar joint range of motion and enhance muscular strength and flexibility, primarily targeting the ankle evertor muscles. Additionally, proprioceptive control training and gait normalization were included in the rehabilitation goals. Physical modalities such as cryotherapy and ultrasound were employed to manage pain and edema, while also providing support for the exercises.

## Discussion

The increasing prevalence of chronic diseases in association with an aging population has been expected to result in over-demand for rehabilitation institutions with a plausible reach of non-sustainable levels. As rehabilitation is being progressively acknowledged as a global health priority, it is of the utmost importance that governments channel more resources to their national health services’ rehabilitation institutions. In our setting, it is not uncommon for patients to wait several weeks to months to initiate a supervised rehabilitation program directed to their functional disability. For certain clinical contexts, such as the case discussed, a long waiting time is unacceptable [2-4]. Thus, alternative high-quality rehabilitative strategies should be sought to maximize functional recovery. To the best of the authors’ knowledge, this is the first study to demonstrate the benefits of a home-based exercise program intervention on functional outcomes in a post-acute case of medial subtalar dislocation.

Home-based exercise programs are usually implemented as a self-management approach for people with chronic diseases. Effectively, these programs constitute a crucial cost-effective intervention adaptable to the patient’s daily schedule by being delivered in the most accessible place for the patient and with the least medical costs [5,6]. Multiple studies have determined the therapeutic benefits of unsupervised home-based programs offering a potential alternative to enhance or replace supervised outpatient rehabilitation programs. A randomized trial by Fleishcman et al. showed unsupervised home exercise was an effective rehabilitation strategy in patients post-total knee arthroplasty, as it provided non-inferior recovery compared to outpatient rehabilitation [7]. Similar results were obtained in low-risk total hip arthroplasty and ankle fracture postoperative patients. Finally, Bassett and Prapavessis demonstrated that home-based interventions were also beneficial for acute injuries when compared to supervised outpatient rehabilitation. The use of cognitive-behavioral strategies, in the latter study, was crucial to prevent poor adherence to treatment [8].

Despite being a known major issue in home-based rehabilitation, our group didn’t find exercise adherence to be concerning in the described case, given the patient’s past as a sportsman and the patient’s demonstration to easily integrate exercise into his daily life. Patient education about the diagnosis and prognosis of subtalar dislocation and understanding the importance inherent to early mobilization and exercise execution were key to assure patient adherence to home-based treatment. Lastly, an additional factor we consider played a part in treatment adherence relates to the acute character of the injury and its immediate functional impact on the patient’s ADL.

The American Orthopaedic Foot & Ankle Society (AOFAS) score is a clinical assessment tool that measures pain, function, and alignment in foot and ankle conditions. We used this score as it provides valuable information for treatment evaluation and decision-making. The score consists of three main components: pain (40 points), function (50 points), and alignment (10 points). Each component assesses specific aspects related to patient-reported outcomes, contributing to a more comprehensive evaluation of the clinical and functional results achieved with our home-based rehabilitation program, as well as the progress made in patients' daily activities participation and quality of life. The benefits obtained with the prescribed program were determined by the notable improvement in functional outcomes, specifically the significant difference of 48 points between the two AOFAS scores and the enhancement in subtalar mobility observed within a two-week timeframe.

At the time of the injury, given the controversy surrounding the period of immobilization and the very low incidence of this injury, a six-week immobilization period was established. Indeed, the optimal duration of immobilization continues to be a subject of debate. Some authors advocate for a period exceeding four weeks to facilitate adequate soft tissue healing, whereas others propose a shorter interval of two to three weeks to be more advantageous in preventing stiffness in the subtalar joint [3]. Lasanianos et al. recommend rehabilitation should commence as early as three weeks following diagnosis [2-4]. In addition, a recent study by Dolan et al. demonstrated that mechanical loading is a critical factor in facilitating soft tissue and bone regeneration which provides additional support for an early rehabilitation approach [9]. Thus, we hypothesize that a shorter immobilization period of less than four weeks following dislocation could have resulted in a higher functional score.

Nonetheless, subsequent to the removal of the cast, the patient was promptly provided with a rehabilitation strategy that prioritized home-based exercises aimed at enhancing flexibility, strengthening, and proprioception. This particular approach played a vital role in the restoration of the patient's subtalar range of motion, improvement of postural control, reduction of pain, and facilitation of increased participation in daily activities, all of which were achieved within a two-week period. It is important to note that the MRI results revealed a tibialis posterior tendon rupture, which may impede further functional progress. To the best of our knowledge, this is the first case reporting such a complication resulting from a closed medial subtalar dislocation. A previous report by Boussakri et al. reported a rupture of this tendon following an open lateral subtalar dislocation [10].

Given the high demand for our hospital's outpatient rehabilitation department, the initiation of a supervised program following a rehabilitation prescription by the physiatrist could entail a minimum waiting period of one month. Therefore, considering the home environment as the most accessible setting, the patient was advised to continue with the temporary management of a home-based exercise program following subtalar dislocation. This recommendation was based on the significant functional benefits observed within a short duration of time.

While recognizing the necessity to monitor and obtain consistent feedback from patients participating in home-based rehabilitation programs, our case report identified a constellation of factors that instilled confidence in our patient's adherence to the prescribed treatment. Adherence predictors such as the patient's intention to actively engage in the program, self-motivation, self-efficacy, and previous history of adhering to physical activity regimens were discerned [6]. We would like to assert that a gap remains in the existing literature regarding well-established scoring systems for self-reported adherence to programs of this nature [5].

## Conclusions

In conclusion, the findings presented in this case report provide evidence supporting the effectiveness of a home-based rehabilitation program as a viable option for managing subtalar dislocation in situations where supervised outpatient rehabilitation services are limited. The documented functional outcomes demonstrate the potential benefits of this approach. In the post-acute phase following subtalar dislocation, initiating early and intensive rehabilitation is imperative to mitigate the risk of long-term complications.
